# Risk of endometrial, ovarian and breast cancer in women with polycystic ovary syndrome: a systematic review and meta-analysis

**DOI:** 10.1093/humupd/dmu012

**Published:** 2014-03-30

**Authors:** John A. Barry, Mallika M. Azizia, Paul J. Hardiman

**Affiliations:** Institute for Women's Health, University College London Medical School, London NW3 2PF, UK

**Keywords:** ovarian cancer, polycystic ovary syndrome, menopause, endometrium, breast

## Abstract

**BACKGROUND:**

Polycystic ovary syndrome (PCOS) is a common condition affecting ∼8% of women. The objective of the present study was to quantify separately the risk of endometrial cancer, ovarian cancer and breast cancer in women with PCOS compared with non-PCOS controls, and quantify separately the risk to women of all ages as well as the risk to premenopausal women.

**METHODS:**

We conducted a systematic review and meta-analysis of observational studies. Studies were eligible for inclusion if they compared women with PCOS to non-PCOS groups for fatal or non-fatal gynaecological cancers. Studies listed in MEDLINE and EMBASE published up to 7 October 2013 in any language were identified, and relevant papers were also searched by hand. Relevant data (for example, study design, source of control data, diagnostic criteria) were extracted and tabulated.

**RESULTS:**

From 698 references, 11 studies (5 of endometrial cancer and 3 each of ovarian and breast cancer) met the inclusion criteria for the meta-analysis (919 women with PCOS and 72054 non-PCOS controls). Using the Mantel–Haenszel method, with fixed or random effects model as appropriate, women with PCOS were at a significantly increased risk of endometrial cancer (odds ratio (OR), 2.79; 95% confidence interval (CI), 1.31–5.95, *P* < 0.008), but the risk of ovarian and breast cancers was not significantly increased (OR, 1.41; 95% CI, 0.93–2.15, *P* < 0.11 and OR, 0.95; 95% CI, 0.64–1.39, *P* < 0*.*78, respectively). However when studies which included women aged over 54 years were excluded from the analysis, the risk for women with PCOS increased further for endometrial cancer (OR, 4.05; 95% CI, 2.42–6.76, *P* < 0.00001), became significantly increased for ovarian cancer (OR, 2.52; 95% CI, 1.08–5.89, *P* < 0.03), but remained non-significant for breast cancer (OR, 0.78; 95% CI, 0.46–1.32, *P* < 0.35).

**CONCLUSIONS:**

This is the first meta-analysis to examine gynaecological cancers in women with PCOS younger than 54 years of age compared with controls of similar age. Current data suggest that women of all ages with PCOS are at an increased risk of endometrial cancer but the risk of ovarian and breast cancer was not significantly increased overall. These results highlight the potential risk of gynaecological cancer morbidities associated with PCOS. However, the available evidence is far from robust and variation in diagnostic criteria for PCOS, associated risk factors (particularly obesity), and selection bias in the studies may have resulted in an exaggeration of the increased risk. Furthermore, women who have PCOS should also be made aware that any increased risk for endometrial cancer must be judged in the context of its relatively low incidence in the general population. A large well-controlled prospective study is required in order to gain a more accurate estimate of the risk of gynaecological cancers in women with PCOS.

**PROSPERO CRD REGISTRATION NUMBER:**

CRD42012003500.

## Introduction

Polycystic ovary syndrome (PCOS) is a very common hormonal disorder affecting ∼5–8% of women of reproductive age (Azziz *et al.*, 2004). It has an insidious onset with a clinical spectrum which includes the classical triad of PCOS: hyperandrogenism, menstrual abnormalities and polycystic ovaries (ESHRE/ASRM, 2004; Azziz *et al.*, 2006; Broekmans *et al.*, 2006). Although originally considered a gynaecologic and endocrine condition, PCOS is now recognized as a multi-system disorder (Solomon, 1999). The discovery of insulin resistance in the 1980s was followed by studies showing an increase of type II and gestational diabetes, and via metabolic syndrome to increased morbidity from coronary heart disease and stroke (Dunaif, 1997; Broekmans *et al.*, 2006; Diamanti-Kandarakis *et al.*, 2007; RCOG, 2007; Shroff *et al.*, 2007).

Concerns that women with PCOS might be at increased risk of cancer date back to the 1940s (Legro, 2007) but this risk is still frequently overlooked by the doctors who care for these women. Any association with malignant disease would be highly important from a public health perspective in view of the high prevalence of PCOS. In practice, this lack of recognition means that women are not advised of this risk, preventative treatment is not offered even to those patients at highest risk and diagnosis of pre-malignant or malignant disease is delayed. In part this lack of recognition can be explained by the relative lack of studies in this field compared with those investigating cardiovascular morbidity in women with PCOS (Hardiman *et al.*, 2003).

The first reports of an association between PCOS and cancer related to endometrial disease (Speert, 1949; Dockerty *et al.*, 1951; Dockerty and Jackson, 1957; Jafari *et al.*, 1978; Gallup and Stock, 1984; Kurabayashi *et al.*, 2003). More recently the possibility of an increased risk of ovarian and breast malignancy has been suggested (Gammon and Thompson, 1991; Schildkraut *et al.*, 1996). At a cellular level there are numerous potential mechanisms which could promote neoplastic disease in women with PCOS, including the prolonged anovulatory state and associated hyperandrogenism with unopposed estrogen action (Key and Pike, 1988; Genazzani *et al.*, 2001). These could increase the risk of cancer through the effect of these hormones on various tissue and organs (Wild *et al.*, 2000; Hardiman *et al.*, 2003). PCOS emerged as a risk factor for endometrial cancer many years ago from a small number of case reports. We previously highlighted the lack of robust evidence to support an association with endometrial cancer (Hardiman *et al.*, 2003) but a recent meta-analysis (Haoula *et al.*, 2012) of pooled data from five comparative studies concluded that women with PCOS were about three times more likely to develop endometrial cancer than other women. Similarly, a population-based case–control study (Schildkraut *et al.*, 1996) raised the possibility of risk of ovarian cancer in women with PCOS, although other studies have not supported this contention (Coulam *et al.*, 1983; Pierpoint *et al.*, 1998). Inconsistent findings also exist regarding the prevalence of breast cancer in women with PCOS (Anderson *et al.*, 1997; Pierpoint *et al.*, 1998).

The cancers discussed in this paper are all highly age-related. The peak incidence for endometrial cancer is ∼90 per 100 000 women (0.09%) at age 70 years. One in eight women develop breast cancer, and this occurs post-menopausally in 80% of cases. Similarly, most cases of ovarian cancer occur post-menopausally (Cancer Research UK, 2013). The last systematic review of endometrial, ovarian and breast cancer risk in PCOS was published in 2009 (Chittenden *et al.*, 2009). The authors identified eight studies to show that women with PCOS were more likely to develop endometrial cancer (odds ratio (OR) 2.70, 95% confidence interval (CI) 1.00–7.29) and ovarian cancer (OR 2.52, 95% CI 1.08–5.89) but not breast cancer (OR 0.88, 95% CI 0.44–1.77). However, their analysis of ovarian cancer included only one study (Schildkraut *et al.*, 1996) and therefore could not provide a meta-analysis of studies of ovarian cancer. No assessment was made of publication bias or the quality of included studies. Chittenden *et al.* (2009) also included three studies that were of polycystic ovaries rather than PCOS (Gammon and Thompson, 1991; Baron *et al.*, 2001; Pillay *et al.*, 2006), making the findings less generalizable to women with a diagnosis of PCOS. Most importantly, the review by Chittenden *et al.* (2009) combined data from women with PCOS of a very wide age range, and the studies included defined PCOS in a variety of ways. Since then Haoula *et al.* (2012) published a systematic review which considered only endometrial cancer and added one more study to those analysed by Chittenden *et al.* (2009). In the light of the Cochrane Collaboration recommendation to update systematic reviews at 2-yearly intervals, we designed the present study to investigate the strength of the association between all three cancers and PCOS (Reeves *et al.*, 2011). In a further development on previous reviews, we investigated the view - which has been expressed but is unproven-that the strength of association of cancer in women with PCOS is influenced by age (Pillay *et al.*, 2006). This is therefore the first report of a separate subgroup analysis of the effect of age, excluding studies that included women over the age of 54 years, who are presumed to be post-menopausal (5 of the 11 studies). A further distinction of this meta-analysis study over previous meta-analyses on this topic is the use of Newcastle Ottawa Quality Assessment Scale (NOS) criteria to assess the methodological quality of included studies.

## Methods

### Data sources and study selection

The guidelines issued by Stroup *et al.* (2000) were followed in the implementation of this meta-analysis. Qualified librarians advised on the search strategy and assisted where papers were difficult to access. The study protocol is available on the Centre for Reviews and Dissemination (CRD) website (registration number CRD42012003500).

### Inclusion and exclusion criteria

Studies that compared cancer events in women with PCOS to non-PCOS controls were eligible for inclusion provided that:

- The study reported the actual number of women from each group who experienced the event and the number who did not experience the event;
- The studies reported other relevant data, e.g. the number of women in each group;

Papers with titles or abstracts that indicated that they were not relevant, for example, animal studies, reviews, or single case studies, were excluded. Papers not published in English were reviewed.

The search replicated that by Chittenden *et al.* (2009). All published studies that assessed the prevalence of both fatal and non-fatal gynaecological cancers were included in the search. The cancer sites included were endometrial, ovarian, and breast cancer. Studies listed in MEDLINE published up to 7 October 2013, and EMBASE from 1980 to 7 October 2013, were identified. Any comparative design was eligible for inclusion (prospective or retrospective, case–control or cohort studies), including studies that used normative population data as a comparison arm of the research design. The following keyword search terms were entered simultaneously: ‘(polycystic ovar* OR polycystic ovary syndrome OR PCOS OR PCO OR Stein-Leventhal) and (breast cancer OR breast carcinoma OR endometrial cancer OR endometrial carcinoma OR ovarian cancer OR ovarian carcinoma OR leiomyosarcoma OR uterine sarcoma OR vulval cancer OR vulvar carcinoma OR cervical cancer OR cervical carcinoma OR vaginal cancer OR vaginal carcinoma OR cervical intraepithelial neoplasia OR CIN)’. A MEDLINE search from 1946 to Week 1 October 2013 retrieved 269 papers. An EMBASE search from 1980 to Week 41 (Week 1 October) 2013 produced 429 results. A hand search of the resulting papers produced no further relevant papers. Authors were contacted where additional information was needed. For example, Iatrakis *et al.* (2006) stated the PCOS diagnosis was confirmed, but did not state which diagnostic criteria was used (in this case, no further data could be obtained).

### Data extraction

Data were collected and coded based on clinical relevance, for example BMI data were collected because of the clinical relevance of BMI to the development of cancer (Renehan *et al.*, 2008). The primary analyses estimated the ORs for cancer in women with PCOS. Sub-analyses were conducted to test for any effect of age and type of PCOS diagnosis.

Information was extracted from each study regarding: (i) characteristics of the participants (age, BMI, ethnicity and method of diagnosis); (ii) the comparison (PCOS compared with controls); (iii) the outcome(s) measured (fatal or non-fatal endometrial, ovarian, or breast cancer, as separate end-points); and (iv) the study design (case–control or cohort). Additional data were extracted in order to assess the methodological quality of each study. The quality assessment criteria were: (i) whether the PCOS diagnosis was sound; (ii) whether selection bias could be ruled out; (iii) whether the control group was selected from hospital or the community; (iv) whether the control group was assessed for PCOS status; (v) whether the PCOS and control group were comparable in terms of age and BMI; (vi) ascertainment of diagnosis; (vii) whether the ascertainment of diagnosis was similar for PCOS and control group; (viii) whether the response rate was similar in the PCOS and control group.

Each article was assessed by authors J.A.B. and P.J.H., and articles that fitted the main criteria (assessing gynaecological cancer in women with PCOS) were accessed. Methodological quality was assessed by J.A.B. and M.M.A. based on the criteria of the NOS for case–control studies (Wells *et al.*, 2011). The Cochrane Non-Randomized Studies Methods Working Group considers the NOS an acceptable tool for assessment of non-randomized studies (Reeves *et al.*, 2011). Inter-rater agreement of NOS scoring was assessed using Cohen's Kappa. A Kappa of 0.41–0.60 is generally considered as demonstrating ‘moderate agreement’ and 0.61–0.80 is ‘substantial agreement’ (Landis and Koch, 1977); The NOS scores of J.A.B. and M.M.A. were in substantial agreement, as shown by the Cohen's Kappa of 0.750 (*P* < 0.00002). Discrepancies were examined and discussed before a final score was agreed upon and assigned.

The raw numbers of events in each study in each of the PCOS and non-PCOS samples from each study were identified and calculated as a common unit, the OR. The OR is the ratio of the odds of the outcomes in each group. The Mantel–Haenszel method was used, with a random effects model in most cases, to generate an OR for all included studies combined. Results were considered statistically significant where the probability value was below the 0.05 threshold. Review Manager statistical software, version 5.2, was used to analyse data.

The outcome measure was a diagnosis of cancer. With the exception of some of the women in one study (Anderson *et al.*, 1997), the women in the included studies were alive at the time of the study measurements; therefore it could be said that the outcome was mainly non-fatal cancer.

## Results

### Eligible articles

**Figure 1:**
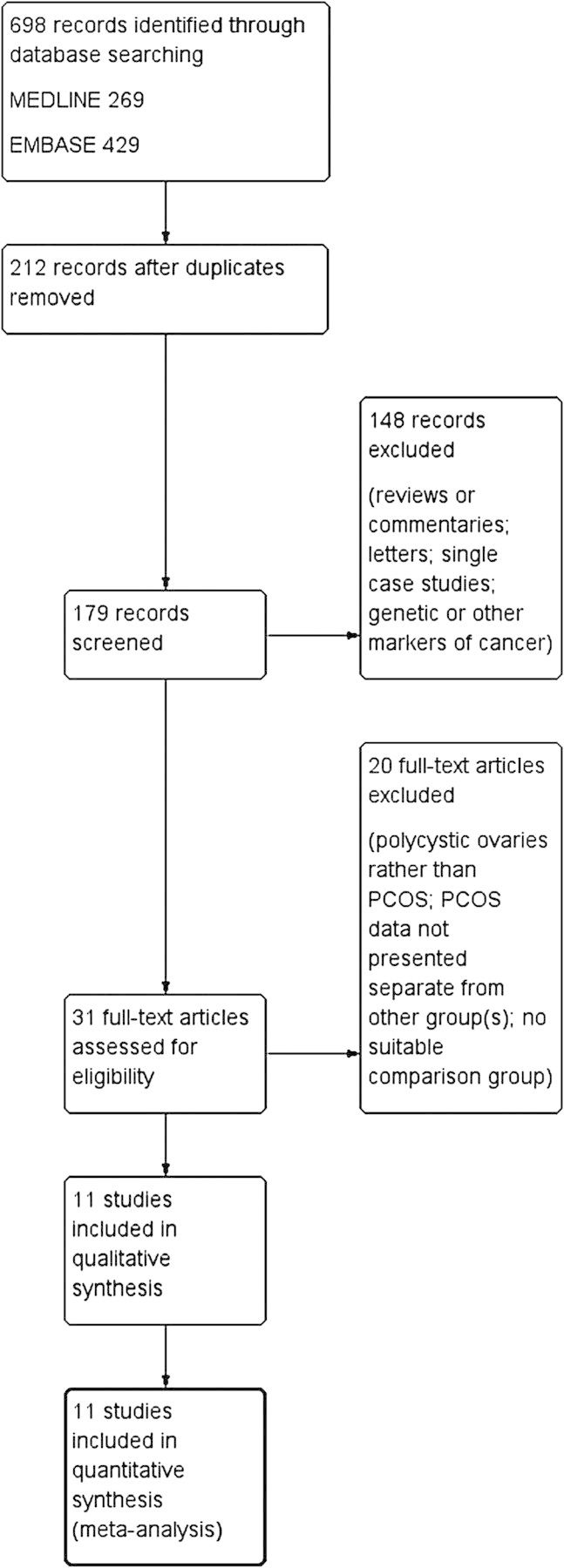
Flow diagram of the literature search for studies of fatal and non-fatal endometrial, ovarian, and breast cancer.

Figure 1 summarizes the search strategy used to identify appropriate studies. Of the 698 initially retrieved, after duplicates were removed the titles and abstracts of 212 records were assessed. A total of 31 full texts were further assessed, including reference sections, and 20 were excluded for various reasons (for example, polycystic ovaries rather than PCOS was identified). Finally, 11 papers met all of the inclusion and exclusion criteria.

The 11 studies that met the inclusion criteria for the meta-analysis (919 women with PCOS and 72 054 non-PCOS controls) are listed in Table I. Most of the studies were: of case–control design, diagnosed cancer histologically, diagnosed PCOS by self-report, did not control for BMI, and consisted mainly of Caucasian patients. Five of the 11 studies included groups of women over the age of 54 years. Because menopause may be clinically relevant to the risk of gynaecological cancers, sub-analyses based on age were carried out.

**Table I:** Characteristics of the 11 studies included in the meta-analysis to assess the risk of endometrial, ovarian and breast cancer in women with polycystic ovary syndrome (PCOS).

Study name	Cancer site	Study design	Diagnostic criteria Ca	Diagnostic criteria PCOS	Age in years [age category]	Controlled for BMI	Ethnic group
Escobedo *et al.* (1991)	Endometrium	Case–control (Dec 1980–1982)	Histologically confirmed Ca infertility patients. Physician diagnosed	Self-report to trained interviewers. Criteria not stated.	All 20–54 (matched age group) [<54]	No	EC cases 92% white, 6% Black; 2% other. Ethnicity of Controls not stated, but selected from same regions as cases
Schildkraut *et al.* (1996)	Ovary	Case–control (Dec 1980– Dec 1982)	Histologically confirmed epithelial Ca. Physician diagnosed	Self-report Stein-Leventhal syndrome or pco from interview. Criteria not stated.	All 20–54 [<54]	No	Not stated, but all from Western USA. Controls chosen by random digit phoning
Anderson *et al.* (1997)	Breast	Cohort (Jan 1986–Dec 1992)	ICD codes 174.0–174.9. Included fatalities.	Self-report. Stein-Leventhal syndrome.	All aged 55–69 [>54]	No significant difference	Mail survey, Iowa, USA
Talamini *et al.* (1997)	Breast	Case–control (1991–1994)	Histologically confirmed Ca. Diagnosed by past history	Self-report. Stein-Leventhal syndrome.	All 20–74 [>54]	Data not shown, but BMI had little impact on ORs	From across Italy.
Niwa *et al.* (2000)	Endometrium	Case–control (1988–1997)	Histologically confirmed primary carcinoma. Physician diagnosed	Clinical data. Diagnosis based on Goldzieher (1981).	All < 40 [<54]	No	100% Japanese
Iatrakis *et al.* (2006)	Endometrium	Case–control (Dec 1992–2004)	Histologically confirmed carcinoma. Physician diagnosed	Self-report. Criteria not stated.	All 43–48 [<54]	No	Not stated, but study is Greek.
Olsen *et al.* (2008)	Ovary	Case–control	New diagnosis of invasive epithelial Ca (*n* = 1276) or borderline malignant tumour (*n* = 315).	Self-report Criteria not stated.	Ca 18–79 Controls age matched ± 5 years [>54]	No, but no significant difference	Not stated except from Australia. Controls from electoral roll.
Zucchetto *et al.* (2009)	Endometrium	Case–control (1992–2006)	Histologically confirmed Ca <1 year; no earlier diagnosis of cancer	Self-report to trained interviewers. Criteria not stated.	All 18–79 [>54]	No	From Pordenone, Milan and Naples, Italy.
Fearnley *et al.* (2010)	Endometrium	Case–control (2003–2007)	Histologically confirmed carcinoma newly diagnosed.	Self-report & criteria not stated.	Ca 18–50 Control <50 [<54]	No	Not stated except from Australia. Controls from electoral roll.
Ghasemi *et al.* (2012)	Breast	Case–control (no dates)	Histologically confirmed primary Ca	Rotterdam, with interview, confirmed by USS.	All 30–51 age matched [<54]	No, but no significant difference	Not stated. Study is Iranian.
Bodmer *et al.* (2011)	Ovary	Case–control (GPRD, 1995–2009)	Ca diagnosis with evidence of Ca-related treatment.	Not stated, but used a diagnosis from GPRD	All 61.2 (SD 13.1) Age matched [>54]	80% BMI matched	∼92% white

Ca, cancer; Rotterdam, Rotterdam diagnostic criteria; GPRD, General Practice Research Database; ICD, International Statistical Classification of Diseases; pco, polycystic ovaries; USS, ultrasound scan; OR, odds ratio; EC, endometrial cancer.

### Methodological quality

Regarding the quality of the studies, in general the NOS scores were moderate. Table II shows that the scores ranged from two to six.

**Table II:** Evaluation of the methodological quality of the 11 studies included in the meta-analysis using an adapted version of the Newcastle-Ottawa Quality Assessment Scale (NOS).

Study	PCOS case definition adequate	Representativeness of PCOS cases	Selection of non- PCOS controls	Definition of non- PCOS controls	Comparability of both groups	Ascertainment of diagnosis	Same ascertainment method for both groups	Non-response rate	NOS score
Escobedo *et al.* (1991)	X	X	X	X	X	*	X	*	2
Schildkraut *et al.* (1996)	X	*	*	*	X	*	*	*	6
Anderson *et al.* (1997)	X	X	*	*	X	*	*	*	5
Talamini *et al.* (1997)	X	X	*	*	X	*	*	*	5
Niwa *et al.* (2000)	X	X	*	*	X	*	*	X	4
Iatrakis *et al.* (2006)	X	X	X	*	X	*	*	X	3
Olsen *et al.* (2008)	X	*	*	*	X	*	*	X	5
Zucchetto *et al.* (2009)	X	X	*	*	*	*	*	*	6
Fearnley *et al.* (2010)	X	*	*	*	X	*	*	X	5
Ghasemi *et al.* (2012)	*	X	X	*	X	*	*	X	4
Bodmer *et al.* (2011)	X	*	*	*	X	X	*	*	5

Note that the evaluation is in regards to being a sound study of PCOS rather than a sound study of cancer.

Note: ‘*’ indicates NOS quality assessment star awarded; ‘X’ indicates that no star was awarded.

### Data analysis

Heterogeneity was assessed using *I*^2^ and χ^2^ statistics. *I*^2^ values of 30% or above were considered likely to indicate moderate heterogeneity and *I*^2^ value >50% indicative of substantial heterogeneity. χ^2^ *P*-values <0.10 were considered to represent significant heterogeneity. Therefore studies showing tolerable levels of heterogeneity (*I*^2^ values <30% and χ^2^ *P*-values >0.10) were analysed using a fixed effects model, and studies showing any greater degree of heterogeneity were analysed using a random effects model.

**Figure 2:**
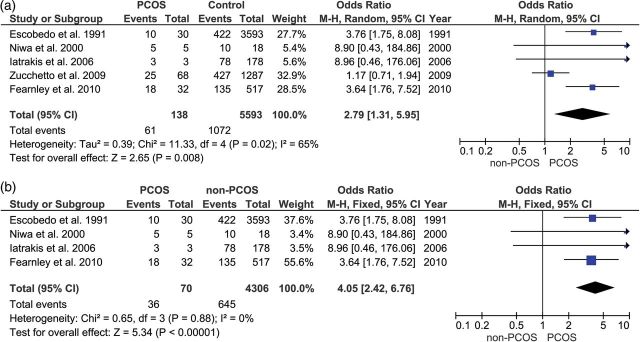
(**a** and **b**) Upper forest plot: endometrial cancer in women with polycystic ovary syndrome (PCOS) compared with controls. Lower forest plot: endometrial cancer in PCOS compared with controls in women under 54 years old.

**Figure 3:**
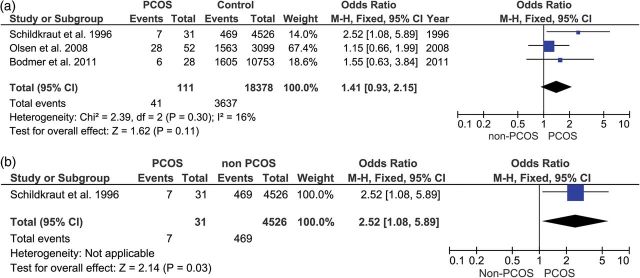
(**a** and **b**) Upper forest plot: ovarian cancer in women with PCOS compared with controls. Lower forest plot: ovarian cancer in PCOS compared with controls in women under 54 years old.

**Figure 4:**
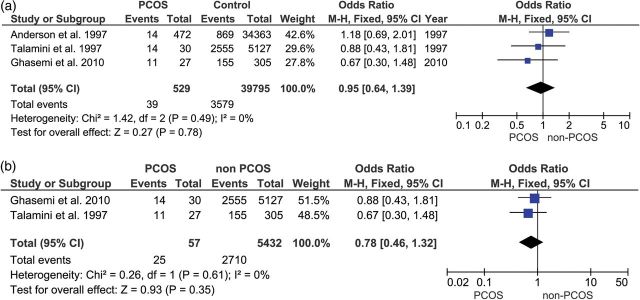
(**a** and **b**) Upper forest plot: breast cancer in women with PCOS compared with controls. Lower forest plot: breast cancer in PCOS compared with controls in women under 54 years old.

Figures 2a and 3a show that the ORs for all five of the studies of endometrial cancer and all three of the studies of ovarian cancer independently showed an increased risk of cancer events associated with PCOS. Figure 4a shows that two of the three studies of breast cancer reported a reduction in risk for women with PCOS. Figure 2b shows that the effect size for endometrial cancer increased when the age of the sample was taken into account. In the only study which reported ovarian cancer in younger women, the risk was significantly higher in women with PCOS (Fig. 3b) but self-evidently it was not possible to perform a meta-analysis, because there was only one study. The risk for breast cancer (Fig. 4b) did not show any significant difference between PCOS and controls, either overall or in the subgroup analysis of younger women.

Table III shows the results of the meta-analysis of the main groups and subgroups. Using the Mantel–Haenszel method, with fixed or random effects model as appropriate, women with PCOS were at a significantly increased risk of endometrial cancer (*P* < 0.008), but not for ovarian cancer (*P* < 0.11), nor breast cancer (<0*.*78). When women aged over 54 years old were excluded from the analysis, the risk for women with PCOS increased further for endometrial cancer (*P* < 0.00001), became significantly increased for ovarian cancer (*P* < 0.03) (although this finding is on data from a single study, so meta-analysis was not possible) and remained relatively unchanged for breast cancer (*P* < 0.35).

**Table III:** Findings of the present meta-analysis of 11 studies, with subgroup analysis based on age of the women (over or under age 54 years).

Disease site	Studies included in analysis	Number of studies	Odds ratio (95% confidence interval)	Z	χ^2^	*I*^2^
Endometrial cancer	All endometrial cancer studies	5	2.79 (1.31–5.95)	2.65 (*P* < 0.008)	11.33 (*P* < 0.02)	65%
	Excluding samples that included women over age 54 years (Zucchetto *et al.*, 2009)	4	4.05 (2.42–6.76)	5.34 (*P* < 0.00001)	0.65 (*P* < 0.88)	0%
Ovarian cancer	All ovarian cancer studies	3	1.41 (0.93–2.15)	1.62 (*P* < 0.11)	2.39 (*P* < 0.30)	16%
	Excluding samples that included women over age 54 years (Olsen *et al.*, 2008; Bodmer *et al.*, 2011)	1	2.52 (1.08–5.89)	2.14 (*P* < 0.03)	*n/a*	*n/a*
Breast cancer	All breast cancer studies	3	0.95 (0.64–1.39)	0.27 (*P* < 0.78)	1.42 (*P* < 0.06)	0%
	Excluding samples of women over age 54 years (Anderson *et al.*, 1997)	2	0.78 (0.46–1.32)	0.93 (*P* < 0.35)	0.26 (*P* < 0.61)	0%

### Publication bias

**Figure 5:**
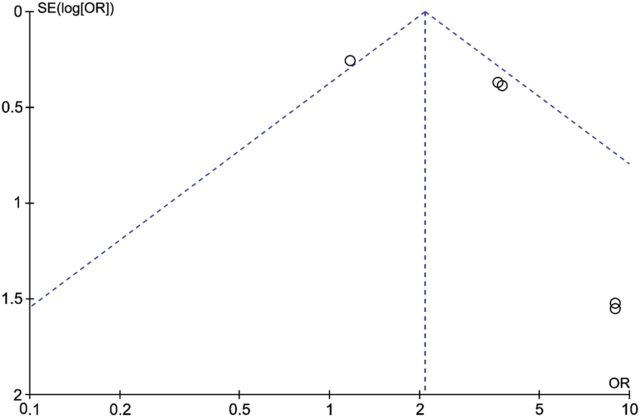
Funnel plot of studies of endometrial cancer in women with PCOS compared with controls.

**Figure 6:**
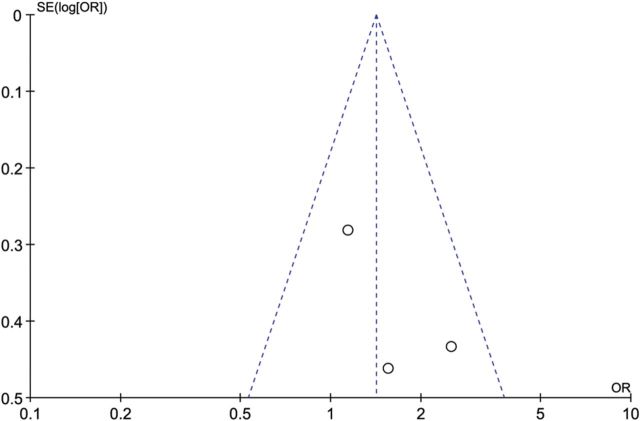
Funnel plot of studies of ovarian cancer in women with PCOS compared with controls.

**Figure 7:**
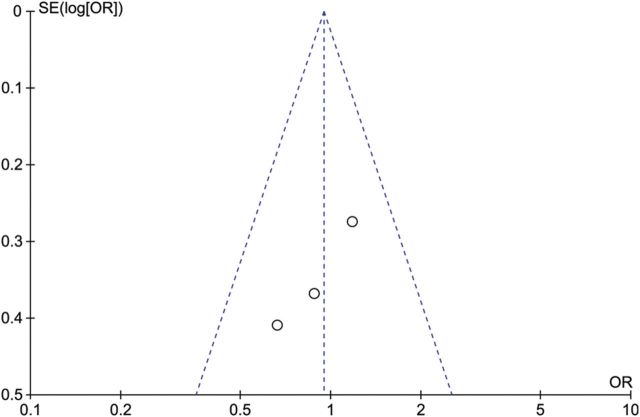
Funnel plot of studies of breast cancer in women with PCOS compared with controls.

To assess publication bias, funnel plots were created (Figs 5–7).

The funnel plot of the findings for endometrial cancer (Fig. 5) is asymmetrical, mainly due to two studies with small sample sizes (Niwa *et al.*, 2000; Iatrakis *et al.*, 2006) (see the lower right of the plot). Because of this asymmetry, publication bias cannot be ruled out. The funnel plots for ovarian and breast cancer studies (Figs 6 and 7) show less asymmetry, though because the number of studies in each plot (*N* = 3) is small, it is difficult to draw firm conclusions regarding publication bias.

## Discussion

This is the first systematic review of gynaecological and breast malignancies in women with PCOS to be published since 2009 and the first ever to separately analyse the risk in premenopausal and post-menopausal women (categorized as women less than and over 54 years of age, respectively) with this syndrome. Our analysis identified two studies published during the 2 years since the last meta-analysis (Haoula *et al.*, 2012). The clinical utility of the two previous meta-analyses (Chittenden *et al.*, 2009; Haoula *et al.*, 2012) is uncertain because in both cases the analyses included patients on the basis of polycystic ovary morphology (PCOM) either on ultrasound or histopathology as well as patients with PCOS: Chittenden *et al.* (2009) had included three studies of PCOM (Gammon and Thompson, 1991; Baron *et al.*, 2001; Pillay *et al.*, 2006) and Haoula *et al.* (2012) included one (Pillay *et al.*, 2006). Around 20% of European women have polycystic ovaries (the prevalence is even higher in some other populations) but approximately two-thirds of these women do not have PCOS (Polson *et al.*, 1988; Clayton *et al.*, 1992). The morbidity associated with PCOS and polycystic ovaries cannot be assumed to be the same and therefore we made the decision not to include studies of women selected on the basis of their ovarian morphology alone. Consequently our results should be more relevant to clinical practice as they reflect the risk amongst our patients rather than asymptomatic women in the general population. However, even after excluding studies based on ovarian morphology, it is possible that variations in diagnostic criteria for PCOS could have introduced selection bias into our analysis; only one study of the 11 included used the Rotterdam criteria (Ghasemi *et al.*, 2012), another two used non-standard criteria (Niwa *et al.*, 2000; Bodmer *et al.*, 2011) and in the remaining eight the syndrome was self-reported so that the criteria were unknown.

Concerns regarding the reliability of PCOS diagnosis are further raised by the prevalence of the syndrome in some of the included studies. For example, Iatrakis *et al.* (2006) found three cases of PCOS in 81 women with endometrial cancer, a prevalence for the syndrome of only 3.7%, but of greater concern is that the prevalence was 0% in the control group. These figures are difficult to reconcile with those found in the general population and cast doubt on the reliability of the data in that study.

Subject to these important caveats, the results of this meta-analysis suggest that women with PCOS of all ages are at an increased risk of endometrial cancer, but the risk of ovarian and breast cancer was not significantly increased overall.

Our analysis of women aged <54 years (in practical terms, premenopausal subjects) confirmed previous suggestions that the risk of endometrial cancer may be even higher in this subgroup of women with PCOS. It is of interest that in the three studies which reported ovarian cancer, the increased risk in women with PCOS was not significant, but the OR was higher and significant in the single study of women aged <54 years. The risk for breast cancer was not significantly different in PCOS compared to the control women overall, or in the subgroup analysis of the two studies of younger women with breast cancer. This finding for endometrial disease is consistent with the observation that post-menopausal women tend to have type II endometrial cancer, which has not been shown to be associated with PCOS, unlike type I (Gadducci *et al.*, 2005).

Overall our results are consistent with previous reviews published on cancer in PCOS (Hardiman *et al.*, 2003; Broekmans *et al.*, 2006) although the addition of further studies to this meta-analysis has added statistical power. A previous meta-analysis (Chittenden *et al.*, 2009) of eight studies found that women with PCOS were more at risk of endometrial cancer and ovarian cancer, but not breast cancer. However, that paper only included one study of ovarian cancer and thus was unable to conduct a meta-analysis for this disease. The meta-analysis of endometrial cancer in PCOS (Haoula *et al.*, 2012) added one study (Fearnley *et al.*, 2010) to those in Chittenden *et al.* (2009), but did not take into consideration another study (Zucchetto *et al.*, 2009), and like Chittenden *et al.* (2009) included one study of polycystic ovaries (Pillay *et al.*, 2006). The present meta-analysis has been able to include two new studies (Ghasemi *et al.*, 2012; Bodmer *et al*, 2011). The finding with respect to endometrial cancer is also in accord with two studies which did not meet the inclusion criteria for our meta-analysis. In a case series of 176 women with endometrial cancer (Dahlgren *et al.*, 1991), the prevalence of hirsutism (but not oligomenorrhoea) was significantly higher in the cases than in the general population; the study was excluded as it did not include any diagnosis of PCOS. In the study by Lunde and Tanbo (2007) of 149 women who underwent ovarian wedge resection for PCOS, the standardized incidence ratio for endometrial cancer was 4.4 but this was not statistically significant; this study was excluded because it did not report the numbers of cancer cases and non-cases in the control group.

As with studies of cardiovascular risk in women with PCOS, assessment of cancer risk is complicated by the possible confounding effects of obesity. High BMI is a recognized risk factor for both endometrial and breast cancer; there is also some evidence of an association with ovarian cancer, although this increased risk may not apply to invasive serous cancers (Webb, 2013). It is therefore possible that our finding of increased risk for endometrial malignancy might result, at least in part, from the increased prevalence of obesity in women with PCOS. In support of this hypothesis, here we found that the risk of ovarian cancer was slightly decreased in studies where BMI was similar in the PCOS and non-PCOS groups. Diabetes is another possible confounding factor in our analysis as this is also associated with an increased risk of endometrial cancer, possibly secondary to hyperinsulinemia, hyperglycaemia and inflammation (Giovannucci, 2007). PCOS shares these risk factors (obesity, diabetes, inflammation, metabolic syndrome, age) and it is unclear whether the increased risk of endometrial cancer is due to individual risk factors (diabetes, obesity) or whether PCOS itself, with its specific metabolic features (such as hyperinsulinism, hyperglycaemia, insulin resistance, hyperandrogenism), increases the risk of cancer. It is possible that the observed association between PCOS and endometrial cancer results from a common inherited genetic variant. Also it is plausible that other factors, such as parity (nulliparous versus multi), age at first pregnancy and use/length of use of hormones (HRT, OCP), may act as confounders.

The range of criteria used to diagnose PCOS is well recognized as a cause for the variation in morbidity associated with this syndrome. This was evident in the studies used in our analysis but this is to some degree understandable because these studies were targeted as risk factors for cancer and were not specifically focused on PCOS. However, unlike the two previous meta-analyses, we excluded data from women with asymptomatic polycystic ovaries. Like the previous meta-analyses, we included self-reported diagnoses of PCOS, and also diagnoses based on unusual criteria (for example, one study was based on Goldzieher's criteria (Niwa *et al.*, 2000)). Some studies predate the accepted Consensus Guidelines (ESHRE/ASRM, 2004) and therefore could be expected not to conform to these criteria (Escobedo *et al.*, 1991; Schildkraut *et al.*, 1996; Anderson *et al.*, 1997; Talamini *et al.*, 1997; Niwa *et al.*, 2000), whilst one study did not specify the diagnostic criteria (Iatrakis *et al.*, 2006). Much of the diagnostic data were self-reported and a diverse range of control cohorts were used (Talamini *et al.*, 1997; Niwa *et al.*, 2000; Baron *et al.*, 2001; Iatrakis *et al.*, 2006). Indeed where studies reported a high risk cancer when compared with age-corrected estimated population incidence, this suggests potential selection bias. Clearly, inclusion of these studies with possible selection biases is a limitation of the present study.

The diversity of the included studies can be clearly seen from Table I. The heterogeneity evident can be summarized as being due to variation in PCOS phenotypes in different ethnicities, diagnostic criteria for PCOS and cohort sizes. To mitigate the effects of heterogeneity we have used a random effects model where appropriate. The CIs for some of the studies crossed zero meaning that no significant risk could be determined, though in the case of endometrial cancer a significant risk was identified (see Table III). Additionally our meta-analysis was limited by the small number of studies suitable for inclusion and their overall quality. We used a comprehensive article and abstract screening process and an extensive three-stage search technique was utilized to maximize article recognition. Although publication bias could not be ruled out (see Figs 5–7), none of the studies included in this meta-analysis were of poor quality, as rated using the NOS for measuring quality assessment (Table II).

On the basis of the available evidence, it is reasonable to conclude that there is an increased cancer risk, at least for endometrium, in women with PCOS. However the other equally important conclusion to be drawn from our systematic review is that the available evidence is far from robust, so that variation in diagnostic criteria for PCOS, associated risk factors (particularly obesity), and selection bias may have resulted in an exaggeration of the increased risk. Women who have PCOS should be made aware that the increased risk we report for endometrial cancer must be judged in the context of the relatively low incidence in the general population (Cancer Research UK, 2013).

The current uncertainty regarding cancer risk in PCOS is not likely to be resolved by additional case–control studies, the design of which allows limited scope for controlling for the effect of different confounding factors and interactions between them. It is imperative that any association between PCOS and cancer is examined further in the context of an appropriately powered prospective longitudinal cohort study. The results of such a study will not only define the risk more clearly, but could facilitate the development of screening of subgroups at greatest risk. This is important because there may be primary interventions, such as lifestyle changes (Birks *et al.*, 2012) and metformin treatment (Landman *et al.*, 2009), that may mitigate this excess risk.

## Ethical approval

Ethical approval was not required for this study.

## Authors' roles

J.A.B. and P.J.H.: study design, database search, collection of data. J.A.B.: analysis of data. J.A.B. and M.M.A.: NOS scoring. J.A.B., M.M.A. and P.J.H.: drafting, interpretation of data and revision of manuscript. Guarantor: P.J.H. All authors had full access to all of the data.

## Funding

This study was conducted without external funding. Departmental funds from the Institute for Women's Health were used to support the authors throughout the study period and manuscript preparation.

## Conflict of interest

All authors have completed the Unified Competing Interest form at www.icmje.org/coi_disclosure.pdf (available on request from the corresponding author) and declare that (i) J.A.B. and P.J.H. have support from UCL for the submitted work; (ii) J.A.B., M.M.A. and P.J.H. have no relationships with companies that might have an interest in the submitted work in the previous 3 years; (iii) their spouses, partners or children have no financial relationships that may be relevant to the submitted work; and (iv) J.A.B., M.M.A. and P.J.H. have no non-financial interests that may be relevant to the submitted work.
